# ADHD, social skills and risky internet use among elementary school children

**DOI:** 10.1186/s13034-025-00926-0

**Published:** 2025-07-10

**Authors:** Songül Derin, Serra Celik, Saliha B. Selman

**Affiliations:** 1 Child and Adolescent Psychiatry, Bezmialem Vakif University, Istanbul, Türkiye; 2 School of Medicine, Bezmialem Vakif University, Istanbul, Türkiye; 3 Department of Psychology, Ibn Haldun University, Istanbul, Türkiye

**Keywords:** ADHD, Social skills, Risky internet use, Elementary school children

## Abstract

**Background:**

Previous studies have established a link between Attention-Deficit/Hyperactivity Disorder (ADHD) and risky internet use (RIU); however, the processes underlying this association remain unclear. This study examines whether a proportion of the association between ADHD and RIU was shared with social skills.

**Methods:**

The sample included 142 children aged 6–12 years (65% female, M = 8.5, SD = 1.7), comprising 71 children diagnosed with ADHD and 71 controls without ADHD. Standardized assessments were administered to measure RIU and social skills. Path analysis was employed to evaluate the association among ADHD, social skills, and RIU. Key demographic variables, including gender, birth timing, age of speech onset, household income, parental education, and number of siblings, were controlled for in the analyses.

**Results:**

An ADHD diagnosis was significantly associated with reduced social skills (β = − 1.68, p < 0.001), and reduced social skills was strongly linked to higher levels of RIU (β = − 0.57, p = 0.004). The direct association between ADHD and RIU was not statistically significant (β = − 0.52, p = 0.169). However, a significant indirect effect was observed, indicating that ADHD-RIU link was shared with reduced social skills (β = 0.96, p = 0.004).

**Conclusions:**

The findings indicate that a significant proportion of the association between ADHD and RIU was shared with social skills, emphasizing the importance of social skills as a potential factor for RIU risk in children with ADHD. Interventions that focus on enhancing social skills may support efforts to address RIU in this population.

## Introduction

Attention-Deficit/Hyperactivity Disorder (ADHD) is a neurodevelopmental disorder affecting around 5% of children worldwide [1]. Alongside its core symptoms of inattention, hyperactivity, and impulsivity, ADHD coincides with challenges such as social deficits and relationship problems [2–4]. Research show that ADHD is associated with higher levels of risky internet use (RIU) [5–7]. Although RIU is not formally classified as a diagnostic category in the Diagnostic and Statistical Manual of Mental Disorders, Fifth Edition (DSM-5) or the International Classification of Diseases, 11th Revision (ICD-11), the term is widely used in developmental research to describe a pattern of excessive preoccupation, loss of control, and functional disruption associated with online activity [8, 9].

Consistent with the Interaction of Person‑Affect‑Cognition‑Execution (I‑PACE) model framework for Internet-use disorders, RIU is conceptualized as an early-stage behavioral risk that can precede more circumscribed conditions such as Internet-gaming disorder [8, 10–12]. Daily internet access before adolescence is now widespread [13, 14]. Surveys show that 68% of children under 2 exceed two hours of daily screen time, 8- to 12-year-olds spend over 5.5 h daily on screens, and 18% of school-age children exhibit problematic internet use [15–18]. Middle childhood offers a key window for early identification and prevention of RIU, as developmental skills are still forming and family influence remains strong [19].

RIU often co-occurs with ADHD [5, 20]. This association is complex and influenced by several factors linked to ADHD. Key traits like impulsivity and emotional dysregulation often correlate with excessive internet use, cyberbullying, and other hazardous online behaviors [2, 4, 21–25]. Cognitive and behavioral dysregulation may further heighten these risks by impairing decision-making and aligning with maladaptive coping [1, 26, 27]. In addition, social isolation and peer rejection can erode self-esteem, coinciding with the increased likelihood of RIU [5, 28, 29]. External stressors, including family conflict, academic failure, and insufficient supervision, are similarly associated with these vulnerabilities, indicating that young people with ADHD may be more prone to RIU [30–32]. Overusing the internet can lead to various adverse outcomes, including social, academic, and personal impairments [33].

Among the key risk factors for RIU are heightened impulsivity and poor social skills [34, 35, 36]. Social skills, encompassing effective communication, emotional expression, and regulation of social interactions, are foundational for children’s psychosocial adjustment [37]. In children with ADHD, deficits in social skills often undermine their ability to interpret social cues and maintain relationships, increasing the risk of isolation, low self-esteem, thereby overlapping with vulnerability to maladaptive online behaviors [1, 2, 4, 23, 34, 38]. These social challenges can create a cycle of emotional distress, aligning with elevated ADHD symptoms and further impairing social functioning [39]. Strengthening social skills might, therefore, be pivotal in addressing such behaviors and supporting in children with ADHD. By focusing on social skills in ADHD-RIU association, this study aims to highlight early risk behaviors that could overlap with more serious concerns.

### The present study

This study examines whether social skills are related to ADHD-RIU association in children, comparing those with ADHD to a well-matched control group to clarify this overlap. We hypothesize that (1) children with ADHD will exhibit poorer social skills than typically developing controls, that (2) poorer social skills will be associated with higher levels of RIU, and (3) a significant proportion of the association between ADHD and RIU will be shared with social skills. To our knowledge, no previous work has specifically examined how social skills intersects with this connection. Understanding this dynamic is crucial for developing targeted interventions that bolster social functioning, potentially reducing RIU and supporting better mental health outcomes in children with ADHD.

## Methods

We report how we determined all data exclusions, sample sizes, manipulations, and measures in the study, consistent with reporting standards for quantitative research [40, 41]. All data, analysis code, and research materials are available by emailing the corresponding author. The current study was not preregistered. Preregistration was not part of our standard research practice at the time of the study's conception. Bezmialem Vakif University Faculty of Medicine's Institutional Review Board approved the study (No: 2023/367).

### Participants

The sample size is discussed in the Discussion section. The sample comprised 142 children aged 6 to 12 years (M = 8.5, SD = 1.7). The case group (n = 71) was recruited through referrals to the Child and Adolescent Psychiatry outpatient clinic at Bezmialem Vakıf University via community resources (e.g., pediatricians, mental health clinics, school personnel, self-referral). The control group (n = 71) was recruited through neighborhood and community schools, family friends of referred children, and other community sources. The Child and Adolescent Psychiatry outpatient clinic is known within the community for providing specialized care in child psychiatry, including comprehensive diagnostic and therapeutic services. The clinic's client base includes children with developmental, behavioral, and emotional issues and typically developing children whose parents consented to participate in research studies. All parents and children provided informed consent/assent to participate in the study.

#### Group assignment

All children and their parents underwent a comprehensive clinical interview to determine eligibility for the study. Seventy-one children who met the following criteria were included in the ADHD group: (1) parent and/or teacher referral to an outpatient ADHD clinic due to reported problems with inattention, hyperactivity, and/or impulsivity (2) a diagnosis of ADHD by a child psychiatrist, using DSM-5 criteria based on the Kiddie Schedule for Affective Disorders and Schizophrenia—Present and Lifetime Version for DSM-5 (K-SADS-PL-DSM-5) interview with both the parent and child, which evaluates the presence and severity of symptoms across both home and school environments. In the patient group, 71 children met the criteria for ADHD-combined type; 6 were comorbid with oppositional defiant disorder, and 3 with anxiety disorder.

Seventy-one children were included in the typically developing group based on the following criteria: (1) absence of any clinical disorder as determined by parent and child interviews using the K-SADS-PL-DSM-5; (2) typical developmental history reported by the mother. Typically, developing children were recruited through neighborhood and community schools, family friends of referred children, and other community sources. Exclusion criteria for the study included a history of (a) significant neurological, sensory, or motor impairments, (b) a history of seizure disorders, (c) psychosis, or (d) intellectual disability.

### Measures

#### Kiddie schedule for affective disorders and schizophrenia-present and lifetime version for DSM-5 (K-SADS-PL-DSM-5)

K-SADS-PL-DSM-5 was utilized to assess psychiatric diagnoses in participants [42]. This semi-structured diagnostic interview is widely recognized for its reliability and validity in evaluating the onset, progression, duration, severity, and impairment associated with both current and past episodes of psychopathology in children and adolescents according to DSM-5 criteria. Trained clinicians conduct the interviews with both the child and their parent or guardian, ensuring comprehensive coverage of affective disorders, psychotic symptoms, anxiety disorders, ADHD and other psychiatric conditions. The psychometric properties of the K-SADS-PL-DSM-5 have been well established within the Turkish population [43].

#### Conners parent rating scale-revised short (CPRS-RS)

The CPRS-RS is a 27-item parent-report questionnaire designed to assess ADHD symptoms in children over the past month [44]. Each item is rated on a Likert scale from 0 to 3, with higher scores indicating greater severity of symptoms. The Turkish adaptation of the CPRS-RS has demonstrated reliable psychometric properties [45]. The CPRS-RS demonstrated excellent internal consistency with a Cronbach's alpha of 0.96.

#### Parent–Child internet addiction test (PCIAT-20)

The PCIAT-20 is a 20-item parent-report questionnaire designed to evaluate signs of internet addiction in children [46]. Each item is rated on a six-point Likert scale from 0 to 5, with higher scores indicating greater severity of symptoms. The Turkish adaptation of the PCIAT-20 has demonstrated reliable psychometric properties [47]. The PCIAT-20 demonstrated excellent internal consistency with a Cronbach’s alpha of 0.94.

#### Social skills evaluation scale (SSES)

The SSES is a 69-item parent-report questionnaire developed to assess social skills in children aged 7–12 years [48]. Each item is rated on a five-point scale, yielding total scores from 69 to 345, with higher scores indicating better social functioning. The Turkish version has demonstrated reliable psychometric properties [48]. The SSES demonstrated strong internal consistency, with Cronbach’s alpha coefficients of 0.84–0.92 for subscales and 0.97 for the total score.

#### Covariates

Gender [49], birth timing [50], total number of siblings [51], age of speech onset in months, household income [52], and parental education levels (mother's and father's education) [53] were selected as covariates based on previous literature linking them to both social skills development and behavioral outcomes in children with ADHD.

### Analysis plan

Descriptive statistics, including means and standard deviations for the study constructs, were summarized in Table 2. All continuous variables had been standardized to enhance interpretability. The analysis began with independent sample t-tests and chi-square tests to compare sociodemographic factors and study variables between the case and control groups. In order to explore the connections among ADHD diagnosis, social skills, and RIU, path modeling was utilized. The main objectives of this investigation were to (1) evaluate both the direct and indirect paths from ADHD diagnosis to RIU, (2) highlight whether a significant proportion of the association between ADHD and RIU had been shared with social skills, and (3) control for any relevant covariates to isolate the main effects of interest.

The hypothesized model estimated the associations among ADHD diagnosis (X), social skills (M), and RIU (Y). In this framework, there was a path from ADHD diagnosis to social skills (a), a path from social skills to RIU (b), and a direct path from ADHD diagnosis to RIU (c'). Additionally, the total association between ADHD diagnosis and RIU (c) was conceptualized as the sum of this direct path (c') and the portion shared with social skills (ab). Any significant indirect findings (ab) were described as evidence that ADHD diagnosis, social skills, and RIU shared variance, recognizing the cross-sectional nature of the data and avoiding any causal inferences.

All statistical procedures were performed using the lavaan package (version 0.6.15) in R (version 4.2.3). Structural Equation Modeling (SEM) with the Maximum Likelihood (ML) estimator and bootstrap-derived standard errors (1000 samples) was applied to strengthen the inference regarding indirect effects. Full Information Maximum Likelihood (FIML) was used to address missing data. Model fit was evaluated using standard indices: a nonsignificant chi-square (χ^2^) implied adequate model fit, while Comparative Fit Index (CFI) and Tucker-Lewis Index (TLI) values over 0.95 signified excellent fit [54]. The Root Mean Square Error of Approximation (RMSEA) and the Standardized Root Mean Square Residual (SRMR) needed to remain under 0.08 to confirm acceptable fit [55, 56]. Monte Carlo simulations were employed to create 95% confidence intervals, thus providing precise estimates of the indirect effects in more sophisticated models [57].

## Results

### Preliminary analysis

#### Demographic differences

The gender distribution between the groups differed significantly (p = 0.02). The case group consisted of 18 females (25%) and 53 males (75%), while the control group had 32 females (45%) and 39 males (55%). The average age of participants in the case group was 8.39 years (SD = 1.54), compared to 8.73 years (SD = 1.85) in the control group (p = 0.2). Birth timing also varied between the groups (p = 0.003), with 12.7% of the case group and 21.1% of the control group born preterm. In contrast, 87.3% of the case group and 67.6% of the control group were born at term, with 11.3% of the control group born post-term. The number of siblings differed significantly (p = 0.02), with most participants in both groups having one or two siblings. The average maternal age was 37.1 years (SD = 5.65) in the case group and 38.8 years (SD = 5.44) in the control group (p = 0.068). Maternal education showed a significant difference (p < 0.001), with 37.1% of mothers in the case group having less than a high school education compared to only 5.6% in the control group. Furthermore, paternal education also differed significantly (p < 0.001), with a higher proportion of fathers in the control group holding university degrees or higher (73%). Household income was significantly higher in the control group (p < 0.001), where 80.3% reported high income, compared to 50.7% in the case group. Demographic information for the two groups is provided in Table 1.

**Table 1:** Child and family characteristics

	Case group (N = 71)	Control group (N = 71)	p-value
Gender			0.02
Male	18 (25%)	32 (45%)	
Female	53 (75%)	39 (55%)	
Age	8.39 (1.54)	8.73 (1.85)	0.2
Birth timing			0.003
Preterm	9 (12.7%)	15 (21.1%)	
Term	62 (87.3%)	48 (67.6%)	
Postterm	0 (0%)	8 (11.3%)	
Number of siblings			0.02
1	20 (28.6%)	9 (12.7%)	
2	39 (55.7%)	37 (52.1%)	
3	10 (14.3%)	15 (21.1%)	
4	1 (1.4%)	7 (9.9%)	
5	0 (0%)	2 (2.8%)	
6	0 (0%)	1 (1.4%)	
Mother age	37.1 (5.65)	38.8 (5.44%)	0.0683
Mother education			< 0.001
Less than high school	26 (37.1%)	4 (5.6%)	
High school	19 (27.1%)	17 (23.9%)	
Associate degree	0 (0%)	15 (21.1%)	
University	25 (35.7%)	28 (39.4%)	
Graduate degree	0 (0%)	16 (21.1%)	
Father age	41.1 (6.34)	42.1 (6.46)	0.363
Father education			< 0.001
Less than high school	29 (41.4%)	4 (5.6%)	
High school	22 (31.4%)	12 (16.9%)	
Associate degree	0 (0%)	3 (4.2%)	
University	19 (27.1%)	41 (57.7%)	
Graduate degree	0 (0%)	11 (15.5%)	
Household income			< 0.001
Low	14 (19.7%)	1 (1.4%)	
Medium	21 (29.6%)	13 (18.3%)	
High	36 (50.7%)	57 (80.3%)	

Continuous variables are presented as mean and standard deviation (M, SD); categorical variables are presented as counts and percentages (N, %); p-values were calculated using independent two-sample t-tests for continuous variables and chi-squared tests of independence for categorical variables

#### Group comparisons

The case group exhibited significantly higher scores across all measures compared to the control group (Table 2). Conners' total scores were higher in the case group (M = 62.4, SD = 12.7) than the control group (M = 26.1, SD = 15.9), indicating more pronounced ADHD-related difficulties (p < 0.001; d = − 2.54, 95% CI [− 2.98, − 2.09]), which represents a very large effect size. Additionally, the case group had significantly higher RIU scores (M = 28.8, SD = 21.9**)** than the control group (M = 20.2, SD = 16.4; p = 0.009; d = − 0.45**,** 95% CI [− 0.78, − 0.11]), reflecting a moderate effect size. This suggests that while children with ADHD engaged in riskier internet behaviors more frequently than their non-ADHD counterparts, the magnitude of this difference was moderate. Children with ADHD also demonstrated significantly lower social skills** (**M = 107, SD = 34.2**)** than the control group (M = 219, SD = 35.1**;** p < 0.001; d = 3.24**,** 95% CI [2.73, 3.74]), representing a large effect size. Only 5 youth with ADHD met or exceeded the overall mean of 163 in social skills, while 66 scored below this threshold, highlighting pervasive social impairments in the ADHD group.

**Table 2:** Comparison of the mean values of ADHD symptoms, RIU and social skills

	Control Group	Case Group	*d*	*p*
Conners Total Score	26.1 (15.9)	62.4 (12.7)	− 2.54	< 0.001
Risky Internet Use Total Score	20.2 (16.4)	28.8 (21.9	− 0.45	0.009
Social Skills Total Score	219 (35.1)	107 (34.2)	3.24	< 0.001

Continuous variables are presented as mean and standard deviation (M, SD); *d* represents Cohen’s *d*, an estimate of effect size; p-values were calculated using independent two-sample *t*-tests for continuous variables

### Primary analysis

 The primary analysis, which was planned a priori, included chi-square (χ^2^ = 23.700, df = 14, p = 0.05), Comparative Fit Index (CFI = 0.96), Tucker-Lewis Index (TLI = 0.94), Root Mean Square Error of Approximation (RMSEA = 0.07), and Standardized Root Mean Square Residual (SRMR = 0.030). These indices suggest that the model demonstrates a good fit to the data. While the main analyses were pre-planned, the specific covariates (gender, birth timing, age of speech onset, total number of siblings, household income, and parental education levels were included in the model) were confirmed after accessing the data and based on relevant literature to better account for potential confounding factors.

The ADHD diagnosis was significantly associated with social skills (a) (β =  − 1.68, 95%CI [− 1.85, − 1.50]) and social skills were significantly associated with RIU (b) (β =  − 0.57, 95%CI [− 0.96, − 0.16]), as these confidence intervals did not include zero. Because all continuous variables were standardized, these coefficients represent effect sizes in standard deviation units. Specifically, a one standard deviation increase in ADHD diagnosis was associated with a 1.68 standard deviation decrease in social skills, and a one standard deviation decrease in social skills was associated with a 0.57 standard deviation increase in RIU.

The direct path from ADHD diagnosis to RIU (c′) was not significant (β = 0.52, 95%CI [− 1.18, 0.77]). Examination of indirect effects indicated that a significant portion of the ADHD–RIU association was shared with social skills (ab) (β = 0.96, 95%CI [0.26, 1.65]). The total effect, including the direct and indirect paths, was also significant (c) (β = 0.44, 95%CI [0.11, 0.76]). These findings suggest that ADHD diagnosis and RIU overlap primarily through social skills rather than via a direct association.

**Fig. 1:**
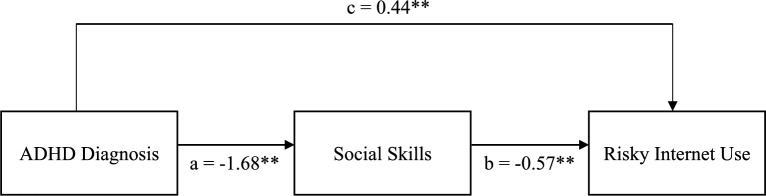
The path model examining the association between ADHD diagnosis, social skills and risky internet use in children

Following the primary analyses, Monte Carlo simulations were performed with 10,000 replications to generate 95% confidence intervals for the parameter estimates. The indirect path of ADHD diagnosis on RIU through social skills was 0.966 (CI: 0.45, 1.49), and the total effect was 0.437 (CI: 0.10, 0.76). See Table 3 for the statistical details. Figure 1 illustrates how ADHD diagnosis, social skills, and RIU are connected through direct and indirect paths.

**Table 3:** Summary of regression analysis

Path	*β*	*p*	95% CI
*Covariates*			
Gender → ADHD Diagnosis	0.13	0.035	[0.01, 0.249]
Birth Timing → ADHD Diagnosis	0.17	0.002	[0.06, 0.267]
Age of speech onset → ADHD Diagnosis	− 0.09	0.006	[− 0.17, − 0.04]
Total number of siblings → ADHD Diagnosis	− 0.16	< 0.001	[− 0.22, − 0.10]
Household Income → ADHD Diagnosis	− 0.07	0.182	[− 0.17, 0.03]
Mother Education → ADHD Diagnosis	− 0.09	0.023	[− 0.17, − 0.01]
Father Education → ADHD Diagnosis	− 0.15	< 0.001	[− 0.23, − 0.07]
*Regression Paths*			
ADHD Diagnosis → Social Skills (a)	− 1.68	< 0.001	[− 1.85, − 1.50]
Social Skills → RIU (b)	− 0.57	0.004	[− 0.96, − 0.16]
ADHD Diagnosis → RIU (c’)	− 0.52	0.169	[− 1.18, 0.25]
*Defined Parameters*			
ADHD on RIU through Social Skills (a*b)	0.96	0.004	[0.26, 1.65]
Total (c) = c’ + a*b	0.44	0.010	[0.11, 0.76]

*β*: standardized regression (path) coefficient; *p*: p‐value, indicating the significance level; 95% CI: 95% confidence interval for β; “ → ” denotes the direction of the predictive path in the structural equation model; a, b, c′, c represents specific path labels; ADHD: Attention Deficit Hyperactivity Disorder; RIU: Risky Internet Use

## Discussion

The present study compared clinically diagnosed 6- to 12-year-olds with ADHD to matched controls to explore how much of the association between ADHD and risky internet use (RIU) is shared with social skills. First, children with ADHD showed substantially poorer social skills than their peers, replicating robust evidence of pervasive interpersonal difficulties in this population [58, 59]. Second, lower social skills co-occurred with more frequent RIU, echoing work linking interpersonal problems to maladaptive online behavior [4, 34]. Third—and most crucial—examination of indirect effects revealed that a significant proportion of the ADHD–RIU relation was shared with social skills, whereas the direct path from ADHD to RIU was not statistically significant. Taken together, these results suggest that social-skill deficits represent an important part of the overlapping variance among ADHD, RIU, and social functioning in middle childhood.

The current pattern aligns with meta-analytic evidence that ADHD is accompanied by elevated screen-based problems [7] and extends those findings by pinpointing social skills as a central locus of overlap. Developmental theories such as the social-compensation model [60–63] posit that youth who find face-to-face interaction effortful may gravitate toward digital settings that appear less demanding. Our data accord with this view: children with ADHD, already prone to social-cue misinterpretation and peer rejection [58, 59], displayed both poorer offline social skills and riskier online habits. Importantly, the magnitude of the shared path was large, reinforcing the practical relevance of interpersonal functioning when considering digital-risk profiles.

Children in the clinical group showed significantly lower total and domain-specific social skills scores compared to controls (all p < 0.001), including deficits in basic social skills, basic and advanced speaking skills, relationship initiation and maintenance, group work, accepting consequences and instruction-giving. Given these widespread deficits, interventions should address both foundational skills (e.g., basic speaking) and higher-order competencies (e.g., relationship maintenance, group work) to strengthen offline social functioning and reduce RIU risk. Although traditional social-skills training for ADHD has shown mixed outcomes [64–66], several complementary strategies can enhance outcomes [67]. Multi-component, school-based social-emotional learning programs that integrate emotion regulation across subjects have shown benefits and may indirectly reduce online risk [68]. Incorporating brief, game-like modules tailored for ADHD may further enhance outcomes [69–71]. Cognitive-behavioral interventions for RIU, effective in adolescents [72, 73], could be adapted for younger children by involving parents [19, 74, 75] and using technology and virtual reality tools [76–78]. Embedding these approaches into interventions may ultimately help mitigate RIU risk [65, 67].

### Strengths and limitations

This study offers several strengths. First, it considers potential confounding factors—such as gender, birth timing, age of speech onset, number of siblings, household income, and parental education—thus providing a clearer view of the connections among ADHD, RIU, and social skills. Next, emphasizing social skills highlights a dimension that may inform clinical strategies. Moreover, conducting the research in Turkiye broadens the global understanding of these associations. Path analysis enabled us to assess direct and indirect effects, offering a more nuanced understanding of the associations among the variables Additionally, Structural Equation Modeling (SEM) with bootstrapped standard errors and Monte Carlo simulations (10,000 resamples) strengthened the robustness of indirect effect estimates, ensuring reliable statistical inference. These methodological strengths collectively enhance the credibility and reproducibility of our findings.

Maxwell et al. [79] raise two key concerns regarding causal interpretations in cross-sectional mediation models: the inability to establish temporal ordering and the risk of misinterpreting statistical associations as evidence of causality. These concerns are relevant to the present study, in which we propose that ADHD symptoms increase the likelihood of RIU via impairments in social skills. While this pathway is theoretically supported, our cross-sectional design does not allow us to confirm the temporal sequence or rule out alternative explanations. That said, the chronic and early-onset nature of ADHD, along with consistent evidence linking ADHD to persistent social skill deficits [58, 59], provides some support for our hypothesized model. Moreover, studies have documented associations between poor social functioning and maladaptive digital behaviors [4, 6], reinforcing the plausibility of our proposed mechanism. Nevertheless, as emphasized by Maxwell et al. [79], these patterns should be interpreted cautiously. Future longitudinal or experimental studies are needed to determine whether ADHD-related social impairments causally contribute to RIU, or whether these associations reflect reciprocal processes or confounding variables.

Fritz and MacKinnon [80] recommend at least 42 participants when using the percentile bootstrap method to detect large indirect effects,our observed path coefficients (a₁ ≈ − 1.68, b ≈ − 0. 57, a × b ≈ 0. 96) meet this threshold, and with a sample of 142, our study is sufficiently powered to large indirect effects. However, given that case and control groups differed on some sociodemographic variables, we included these covariates in the path model, which may have reduced the effective power relative to the original estimates. As such, while the mediation effect size is large, the study may not have been adequately powered to detect more nuanced or small-magnitude effects, and findings should be interpreted with appropriate caution. Additionally, recruiting from a clinical setting may influence the generalizability of the findings, as the sample may not fully represent the broader population. Future studies with larger and more diverse samples could enhance the robustness and generalizability of the results.

The reliance on self-report measures may introduce bias. Self-report measures are subject to social desirability and recall biases, which can affect the accuracy of the reported information. Another potential limitation is the exclusive reliance on parent-report measures, which may introduce shared method variance and inflate associations among ADHD symptoms, social skills, and RIU. Although diagnostic evaluations included the K-SADS-PL-DSM-5 and the parent-reported Conners, the absence of a validated teacher-report measure—relying instead on informal phone interviews—limits cross-informant verification. Incorporating multi-informant approaches, such as standardized teacher ratings or objective app-use data, would strengthen measurement validity and reduce mono-informant bias. The study also lacks a comprehensive measurement of parental psychopathology symptoms as it may influence symptom reporting, although only thirteen mothers and twelve fathers in the case group and seven mothers and no father in the control group reported a psychiatric condition.

In conclusion, within a rigorously assessed cohort of primary-school children, ADHD, poor social skills, and RIU were tightly interwoven. Social-skill deficits accounted for most of the shared variance between ADHD and RIU, underscoring interpersonal functioning as a promising target for prevention efforts. Addressing both ADHD symptoms and social skill deficits could lead to more effective interventions for reducing RIU in this population.

## Data Availability

The data supporting the findings of this study are available on request from the corresponding author.
